# Novel endolysin from *Streptococcus iniae*-Specific Prophage Selectively Inhibits Target Bacteria

**DOI:** 10.4014/jmb.2508.08038

**Published:** 2025-12-29

**Authors:** JoonBeom Moon, Hanbeen Kim, Suryang Kwak, Jakyeom Seo

**Affiliations:** 1 Department of Animal Science, Life and Industry Convergence Research Institute, Pusan National University, Miryang 50463 Republic of Korea; 2 Faculty of Land and Food Systems, The University of British Columbia, Vancouver, BC Canada V6T 1Z4; 3 Department of Bio and Fermentation Convergence Technology, Kookmin University, Seoul 02707, Republic of Korea

**Keywords:** Aquaculture, *Streptococcus iniae*, Endolysin, Bacteriophage

## Abstract

*Streptococcus iniae* is a gram-positive, spherical- or ovoid-shaped, facultative anaerobic bacterium and is one of the major species causing streptococcosis, resulting in economic losses in aquaculture. Endolysins, peptidoglycan hydrolases produced by bacteriophages, are emerging as replacements for antibiotics due to their specific lytic activity against pathogens. This study aimed to develop a novel endolysin, SinLys1930, that specifically targets and kills *S. iniae*. The molecular and structural characteristics of SinLys1930 were predicted based on bioinformatic approaches. The lytic activity of SinLys1930 was evaluated against *S. iniae* KCTC 3657 under various conditions, including different dosages, pH levels, temperatures, NaCl concentrations, and metal ions, to identify the optimal conditions, and its effectiveness was further tested in sterilized seawater. The conserved domain analysis revealed that SinLys1930 possesses two enzymatically active domains (NlpC/P60 and glucosaminidase superfamilies) with two cell wall-binding domains (CW-7 superfamily) positioned between them. The lytic activity of SinLys1930 was highest at pH 6.0 to 6.5 and temperatures between 16 and 37°C, and it was maintained even under high NaCl concentration. SinLys1930 reduced the optical density of *S. iniae* in sterilized seawater by approximately 60% after incubation for 1 h. Therefore, SinLys1930 could potentially serve as an alternative to antibiotics for preventing streptococcosis caused by *S. iniae* in the aquaculture industry.

## Introduction

Aquaculture is a rapidly growing food production industry worldwide and plays a fundamental role in supplying protein food sources. Due to the growing emphasis on the increasing world population, which is projected to reach 9.73 billion people by 2064 [1], aquaculture industry production is estimated to increase to 109 million tons by 2030 [2]. Therefore, to meet the rising consumer demand, implementing intensive farming systems that boost fish production and enhance feed efficiency is advisable. Nonetheless, intensive farming systems can also create favorable conditions for pathogens, resulting in increased transmission and frequency of infections, as well as promoting the survival and virulence of these pathogens [3]. Several pathogenic diseases are prevalent in aquaculture, including vibriosis (*Vibrio alginolyticus*, *V. fluvialis*, and *V. parahaemolyticus*), aeromoniasis (*Aeromonas hydrophila* and *A. salmonicida*), edwardsiellosis (*Edwardsiella ictaluri* and *E. tarda*), and streptococcosis (*Streptococcus iniae*, *S. agalactiae*, and *S. parauberis*). These pathogenic diseases not only result in high mortality rates and economic losses in the aquaculture industry but also facilitate the transmission of zoonotic bacteria, posing a critical threat to human health [4, 5].

Streptococcosis is a re-emerging bacterial disease in the aquaculture industry that threatens its development worldwide. Although various *Streptococcus* species, including *S. iniae*, *S. agalactiae*, *S. parauberis*, *S. uberis*, and *S. dysgalactiae*, are known to cause streptococcosis [5], *S. iniae* and *S. agalactiae* are the primary species responsible for streptococcosis in aquaculture. *S. iniae* is a gram-positive, spherical- or ovoid-shaped, facultative anaerobic bacterium that is β-hemolytic on 5% sheep blood agar [5]. *S. iniae* infections have been reported in various fish species globally, including olive flounder (*Paralichthys olivaceus*), salmon (*Salmo salar*), gray mullet (*Mugus cephalus*), barramundi (*Lates calcarifer*), yellowtail (*Seriola quinqueradiata*), and red drum (*Sciaenops ocellatus*)[6]. Fish infected with *S. iniae* exhibit various symptoms such as septicemia, meningitis, panophthalmitis, exophthalmia, corneal opacity, and lethargy, resulting in high levels of mortality and morbidity [6].

Various antibiotics, including florfenicol, erythromycin, doxycycline, and oxytetracycline, have been used to control streptococcosis in aquaculture [5]. However, the use of antibiotics to control bacterial infections in fish has been limited due to several factors, including the high cost of frequent treatments, the increasing prevalence of antibiotic-resistant bacteria, and the potential presence of antibiotic residues in fish products [5]. In this regard, various approaches, such as phage therapy, vaccinations, biosurfactants, antimicrobial peptides, and probiotics, have been investigated to replace antibiotics [7].

Endolysins are peptidoglycan (PG) hydrolases that break down the PG layer at the end of the lytic cycle of bacteriophages [8]. Endolysins have been receiving increasing attention as an alternative to antibiotics due to their rapid lytic activity, high host specificity, and lack of evidence of resistance or residuals in the body [9]. Recently, several studies reported the successful development of recombinant endolysins against *V. parahaemolyticus*, *V. alginolyticus*, *A. hydrophila*, and *Acinetobacter baumannii* and successfully demonstrated their inhibitory effects against target bacteria in the fish industry [10-12].

The conventional method for developing novel recombinant endolysins includes screening suitable bacteriophages capable of killing target bacteria and determining the gene sequence encoding the endolysin in the selected bacteriophages [10, 13]. These processes are typically time-consuming and labor-intensive, requiring extensive culturing, purification, enrichment, and DNA manipulation to isolate endolysin genes. To address this limitation, several studies have shown that putative endolysin genes, identified through bioinformatics tools, can facilitate the development of recombinant endolysins, eliminating the need for labor-intensive bacteriophage screening [14, 15]. Therefore, this study aimed to develop a novel endolysin against *S. iniae* utilizing bioinformatics tools and to assess its optimal conditions for lytic activity under various environments, including sterilized seawater.

## Materials and Methods

### Bacterial Strains and Growth Conditions

The bacterial strains used in this study were purchased either from the Korean Collection for Type Cultures (KCTC, Republic of Korea) or the Korean Culture Center of Microorganisms (KCCM, Republic of Korea)(Table S1). *S. iniae* KCTC 3657 was used as the indicator strain to assess the optimal lytic conditions for the recombinant endolysin developed in this study. *Escherichia coli* cloning strain DH5α and expression strain BL21 (DE3) were aerobically grown at 37°C in Luria–Bertani (LB) broth (Difco Laboratories Inc., USA). *S. iniae*, *S. parauberis*, *Bacillus subtilis*, and *Enterococcus faecalis* were cultured aerobically at 37°C in brain heart infusion (BHI) broth (Difco Laboratories Inc.), whereas other streptococcal species were grown anaerobically.

### Cloning, Overexpression, and Purification of Recombinant SinLys1930

Genome sequences of *S. iniae* were obtained from the National Center for Biotechnology Information (NCBI) genome browser and annotated using the Rapid Annotations using Subsystems Technology (RAST) server [16]. All putative genes related to the lysis modules of bacteriophages were gathered and analyzed using the NCBI conserved domain database (CDD) [17]. Based on the conserved domain analysis, a putative endolysin gene was identified and designated SinLys1930. The SinLys1930 nucleotide sequence was chemically synthesized following codon optimization and subsequently cloned into the pET28b expression vector (Novagen Inc., USA), which includes an N-terminal hexa-histidine tag (6×His tag) sequence, by Bionics Inc. (Republic of Korea). The cloned plasmid was transformed into competent *E. coli* BL21 (DE3) cells which were cultured in LB broth until reaching an optical density of 0.4 at 600 nm (OD_600nm_). Thereafter, 1 mM isopropyl β-D-1-thiogalactopyranoside was added to induce protein expression, and the culture was incubated for an additional 18 h at 16°C. The cells were then harvested, resuspended in lysis buffer (50 mM NaH_2_PO_4_, 300 mM NaCl, and 10 mM imidazole, pH 8.0), and lysed on ice using sonication (KYY-80; Korea Process Technology Co., Ltd., Republic of Korea). After centrifugation at 10,000 ×*g* for 15 min, the supernatant was collected and further purified using Ni-NTA resin (Qiagen GmbH, Germany), following the manufacturer’s instructions. The purified SinLys1930 was subjected to sodium dodecyl sulfate-polyacrylamide gel electrophoresis (SDS-PAGE) and then pooled and dialyzed against elution buffer (50 mM NaH_2_PO_4_ and 300 mM NaCl, pH 8.0).

### Prediction of Structural Aspects of SinLys1930

The secondary structure of SinLys1930 was predicted by submitting its amino acid sequence to the JPred4 server [18]. The amino acid sequence of SinLys1930 was submitted to the ColabFold, which implements AlphaFold2 with MMseq2, to predict its protein structure (https://colab.research.google.com/github/sokrypton/ColabFold/blob/main/AlphaFold2.ipynb) [19]. The predicted three-dimensional structure with the highest predicted local distance difference test score was visualized using UCSF ChimeraX (https://www.cgl.ucsf.edu/chimerax, version 1.3) [20].

### Determination of SinLys1930 Antibacterial Activity

The antibacterial effect of SinLys1930 was evaluated using a colony forming unit (CFU) reduction assay, following the method described previously [21]. Briefly, *S. iniae* was cultured to an OD_600nm_ of 0.8 to 1.0, harvested, and resuspended in an equal volume of sodium phosphate buffer (pH 6.0). Subsequently, 500 μl of SinLys1930 was added to 4.5 ml of the resuspended bacterial culture (final concentration, 1.96 μM) and incubated for 2 h at 25°C. The resulting mixture was serially diluted in 10-fold increments and plated onto BHI agar plates. After 24 h of incubation at 37°C, CFUs was counted to determine the number of viable cells. The above tests were performed in triplicate, and the mean CFU values were calculated. The final CFU was determined by multiplying the average number of colonies by the dilution factor.

### Assessment of SinLys1930 Antibacterial Activity

The lytic activity of SinLys1930 was assessed as the reduction rate at OD_600nm_ [15]. Briefly, to evaluate dose-dependent responses, *S. iniae* was cultured until reaching an OD_600nm_ of 0.8 to 1.0, then harvested and resuspended in an equivalent volume of 50 mM sodium phosphate buffer (pH 6.0). Serial dilutions of endolysin (20 μl, final concentrations ranging from 0.06 to 3.91 μM) were added to 96-well cell plates (SPL Life Sciences Co., Ltd., Republic of Korea) containing 180 μl of the cell suspensions and incubated at 37°C. OD_600nm_ readings were taken every 10 min for one hour using an iMark microplate reader (Bio-Rad Laboratories, USA). To determine the optimal temperature for lytic activity, SinLys1930 (final concentration, 1.96 μM) was tested at 4, 16, 25, 37, 45, and 55°C. The optimal pH was assessed by resuspending *S. iniae* KCTC 3657 in 50 mM sodium acetate buffer (pH 5.0 and 5.5) and 50 mM phosphate buffer (pH 6.0 to 8.0). The impact of NaCl concentration on SinLys1930 activity was investigated by adding NaCl at concentrations of 50, 100, 200, 400, and 800 mM to the optimal pH buffer. The effects of divalent cations were assessed following the method previously described [15]. Briefly, SinLys1930 (19.6 μM) was incubated with 5 mM ethylenediaminetetraacetic acid (EDTA) at 25°C for 30 min to chelate divalent cations attached to the endolysin. Thereafter, EDTA was replaced with an empirically determined buffer at the optimal pH using an Amicon Ultra-4 centrifugal filter (10 kDa) (Merck KGaA, Germany). The lytic activity of the endolysins was evaluated after incubation with EDTA, 10 mM CaCl_2_, MgCl_2_, MnCl_2_, or ZnCl_2_. All experiments were performed in triplicate.

The reduction rate for the buffer control and SinLys1930 treatment groups was calculated using the following formula:

Reduction rate = (OD_600nm-initial_ – OD_600nm-1 h_) / OD_600nm-initial_ × 100

The OD_600nm-initial_ refers to the initial optical density at 600 nm, and OD_600nm-1 h_ refers to the optical density at 600 nm measured 1 h after the treatment.

The lytic activity was calculated as the difference in reduction rate between the treatment and the control groups:

Lytic activity (%) = (Reduction rate_treatment_ – Reduction rate_control_)

### Spectrum of Lytic Activity

The lytic spectrum of SinLys1930 was tested on *S. parauberis*, *Streptococcus sanguinis*, *Streptococcus mutans*, *Streptococcus alactolyticus*, *Streptococcus gallolyticus* subsp. *pasteurianus*, *B. subtilis*, *E. faecalis*, *E. coli* BL21, and *E. coli* DH5α. Briefly, the bacteria were grown until they reached an OD_600nm_ of 0.8 to 1.0, then harvested and resuspended in an equivalent volume of 50 mM sodium phosphate buffer (pH 6.0). To assess the lytic activity spectrum of SinLys1930, 20 μl of SinLys1930 was added to 96-well cell plates containing 180 μl of each bacterial cell suspension (final concentration, 1.96 μM). The mixtures were incubated at 25°C, and the decrease in OD_600nm_ was measured after 1 h to determine lytic activity.

### Lytic Activity of SinLys1930 in Seawater

To evaluate the lytic activity of SinLys1930 in seawater, seawater was collected from the Southern Sea in South Korea and autoclaved at 121°C for 15 min to sterilize the samples. The sterilized seawater underwent centrifugation at 20,000 ×*g* for 15 min, followed by filtration through a 0.2-μm micro-filter to collect the supernatant (Whatman, UK). *S. iniae* cultures were grown to an OD_600nm_ of 0.8 to 1.0, then harvested and resuspended in an equal volume of sterilized seawater. To standardize the metal ion environment and support enzymatic activity, SinLys1930 was pre-incubated with 10 mM MgCl_2_, based on the metal ion characterization results. MgCl_2_ treated SinLys1930 (20 μl, final concentration, 1.96 μM) was added to 96-well cell plates containing 180 μl of each bacterial suspension. The mixtures were incubated at 25°C, and OD_600nm_ readings were taken after 1 h using an iMark microplate reader (Bio-Rad Laboratories).

### Statistical Analysis

Statistical analysis to assess differences in SinLys1930 lytic activity under varying conditions was performed using R software (R version 4.1.1; R Foundation for Statistical Computing, Austria). Due to the non-normal distribution of residuals, a non-parametric Kruskal–Wallis test was conducted using the kruskal.test function. When a significant effect was detected, group comparisons were made using Dunn's multiple comparison test via the dunnTest function in the FSA package. All *P*-values were adjusted according to the Benjamini–Hochberg false discovery rate correction. Statistical significance was declared at *P* < 0.05.

## Results

### Structural Analysis of SinLys1930

**Fig. 1:**
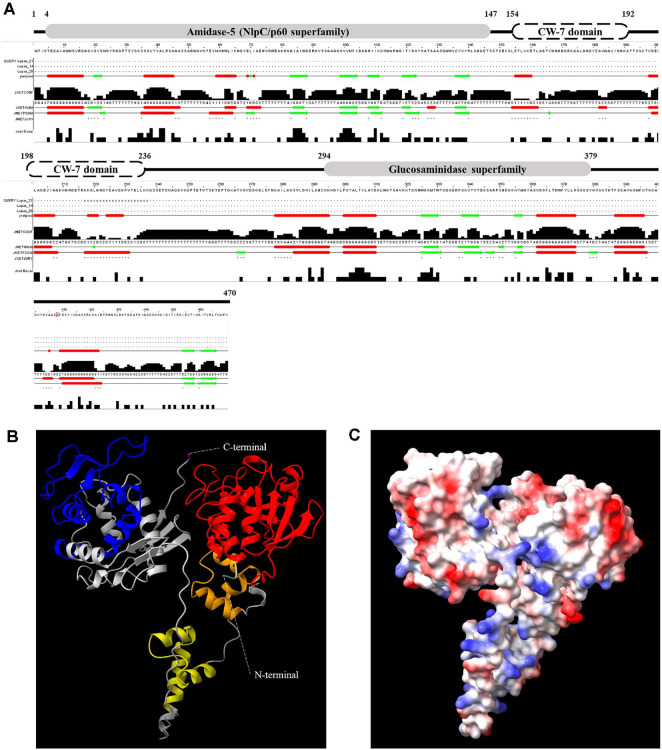
Structural characteristics of SinLys1930. (**A**) Conserved domains and secondary structures of SinLys1930. Gray squares represent enzymatically active domains at the N- terminus (NlpC/p60 superfamily) and C-termius (glucosaminidase superfamily). White squares represent the cell wall-binding domain (CW-7 superfamily). (**B**) *In silico* ribbon diagram of SinLys1930 predicted by AlphaFold2 using the ColabFold notebook (https://colab.research.google.com/github/sokrypton/ColabFold/blob/main/AlphaFold2.ipynb). The ribbon form shows the NlpC/p60 superfamily (red), CW-7 superfamily (orange and yellow), and glucosaminidase superfamily (blue) of SinLys1930. (**C**) Electrostatic potential of SinLys1930 determined by ChimeraX (https://www.cgl.ucsf.edu/chimerax,version 1.3). The positive and negative potentials are shown in blue and red, respectively.

Putative genes associated with the bacteriophage lysis module were initially retrieved from genome sequences of *S. iniae* after annotation *via* the RAST server. Among the seven putative genes identified, we selected SinLys1930 for further analysis due to its complete complement of both enzymatically active domains (EADs) and cell wall-binding domains (CBDs) components. Amino acid sequence analysis using the NCBI CDD showed that SinLys1930 was predicted to be a 470-amino acid protein with a modular design similar to most endolysins from bacteriophages targeting gram-positive bacteria. SinLys1930 had two distinct EADs at the N- and C-termini belonging to the NlpC/p60 (amidase-5, pfam05382, *e*-value = 1.73 × 10^-78^) and glucosaminidase superfamilies (cl29459, *e*-value = 8.34 × 10^-04^), respectively. Two CBDs of the CW-7 superfamily (cl07020, *e*-value = 3.08 × 10^-10^; Cpl-7, smart01095, *e*-value = 3.10 × 10^-14^, respectively) were observed in the middle of the sequences (Fig. 1A). The NlpC/p60 superfamily was folded with three α-helices and six β-sheet strands, while the glucosaminidase superfamily was folded with two α-helices and three β-sheet strands. The CBDs (CW-7 superfamily) of SinLys1930 were predicted to have differing structures (two and three α-helices). The three-dimensional model of SinLys1930 was predicted using AlphaFold2 in the ColabFold notebook, which displayed the highest predicted local distance difference test score and was visualized in ribbon and Connolly surface forms in ChimeraX (version 1.3) (Fig. 1B and 1C). The EADs and CBDs of SinLys1930 were predicted to be folded independently.

### Characterization of SinLys1930

**Fig. 2:**
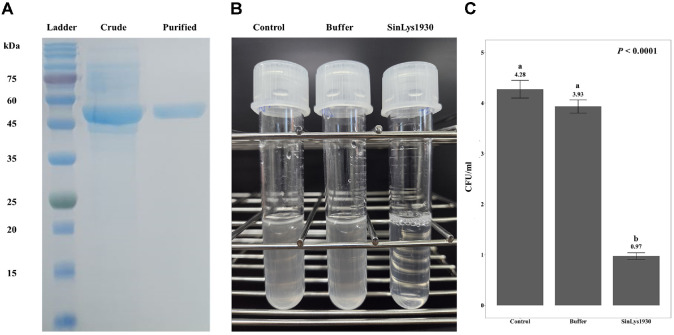
Lytic activity of purified SinLys1930 against *Streptococcus iniae* KCTC 3657. (**A**) Purified SinLys1930 resolution on 15% SDS-PAGE. Ladder, stained protein molecular weight markers; Crude, crude form of SinLys1930; Purified, purified form of SinLys1930. (**B**) Lysis of *S. iniae* by the purified SinLys1930. Control, *S. iniae* resuspended in sodium phosphate buffer (10 ml); Buffer, *S. iniae* resuspended in sodium phosphate buffer (9 ml) with the addition of 1 ml recombinant protein elution buffer; SinLys1930, *S. iniae* resuspended in sodium phosphate buffer (9 ml) with the addition of 1 ml SinLys1930 (final concentration, 1.96 μM). (**C**) Assessment of the lytic activity of endolysin SinLys1930 by colony forming unit reduction assay (n = 4). Groups that do not share a superscript letter (a and b) differ significantly (*P* < 0.0001, Dunn's multiple comparison test with Benjamini-Hochberg false discovery rate correction).

Recombinant SinLys1930 was successfully produced in *E. coli* BL21 (DE3) and purified using nickel affinity chromatography with an N-terminal 6×His tag. The major band of soluble SinLys1930 endolysin was observed above the 45-kDa marker on SDS-PAGE after purification (Fig. 2A), which aligns with its theoretical molecular weight (51.1 kDa).

**Fig. 3:**
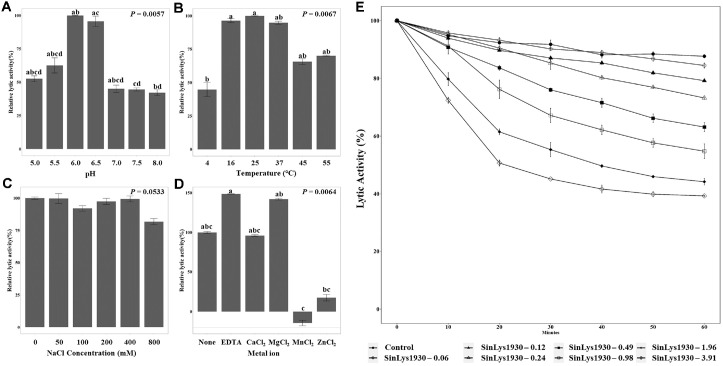
Lytic activity of SinLys1930 against *Streptococcus iniae* KCTC 3657 under varying (A) pH, (B) temperature, (C) NaCl concentration, (D) metal ion conditions, and (E) dosages. In Fig. 3C, no significant differences were observed under NaCl conditions (*P* = 0.0533). In Fig. 3D, None, purified SinLys1930; EDTA, purified SinLys1930 after incubation with 5 mM ethylenediaminetetraacetic acid (EDTA); Other metal cation treatments (Ca^2+^, Mg^2+^, Mn^2+^, and Zn^2+^), EDTA-treated SinLys1930 after adding 10 mM each metal ion. In Fig. 3E, serial dilutions of SinLys1930 (20 μl, final concentrations ranging from 0.06 to 3.91 μM) were added to 180 μl of the cell suspensions and incubated at 37°C. Data are shown as means ± standard deviation of biological triplicates. Groups sharing at least one superscript letter (a-d) are not significantly different from each other, whereas groups that do not share any letters differ significantly (*P* < 0.05, Dunn's multiple comparison test with Benjamini-Hochberg false discovery rate correction).

Subsequently, we evaluated the lytic activity of SinLys1930 using *S. iniae* KCTC 3657 as the standard target strain. The addition of SinLys1930 reduced the turbidity of *S. iniae* KCTC 3657 resuspended in sodium phosphate buffer (pH 6.0) (Fig. 2B). Similarly, the CFU of *S. iniae* KCTC3657 decreased with SinLys1930 treatment after 24 h of incubation (Fig. 2C). The viable cell count for the control and buffer group was each 4.28 × 10^10^ CFU/ml and 3.93 × 10^10^ CFU/ml, respectively, while the SinLys1930 treatment group showed a significant reduction in viable cell number, with a CFU count of 9.7 × 10^9^ CFU/ml. Lytic activity was the highest at pH 6.0 (Fig. 3A) and 25°C (maintained between 16 and 37°C) (Fig. 3B). The lytic activity of SinLys1930 was not affected by the addition of NaCl (*P* = 0.0533, Fig. 3C). Adding EDTA and Mg^2+^ increased the lytic activity of SinLys1930 by approximately 50%, whereas Mn^2+^ and Zn^2+^ decreased the lytic activity significantly (Fig. 3D). As the concentration of SinLys1930 increased, the viability of *S. iniae* correspondingly decreased. The addition of 3.91 μM SinLys1930 resulted in approximately 60% lytic activity relative to the control after 60 min of incubation (Fig. 3E).

### Lytic Spectrum of SinLys1930

**Fig. 4:**
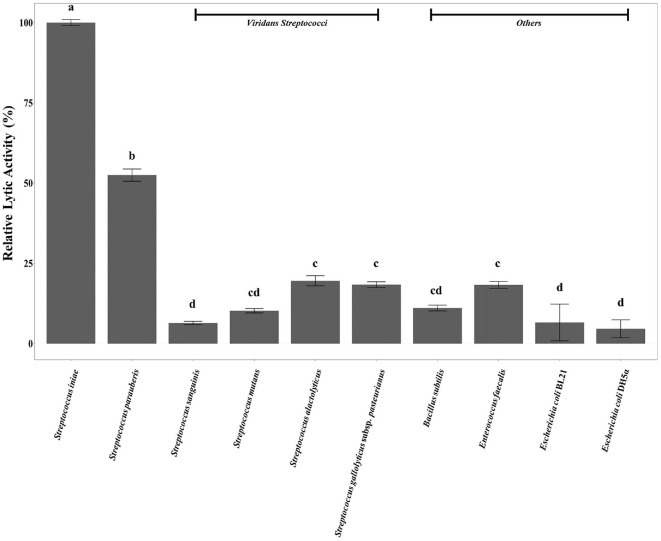
Lytic activity spectrum of SinLys1930. Relative lytic activity (%) is defined as lytic activity against each bacterium divided by the lytic activity against *Streptococcus iniae* KCTC 3657. All lytic activity tests were conducted under optimal conditions for SinLys1930 (final concentration, 1.96 μM; pH 6.0; 25°C; with Mg^2+^ treatment). The error bars denote standard deviation of biological triplicates. Groups sharing at least one superscript letter (a-d) are not significantly different from each other, whereas groups that do not share any letters differ significantly (*P* < 0.05, Dunn's multiple comparison test with Benjamini-Hochberg false discovery rate correction).

**Fig. 5:**
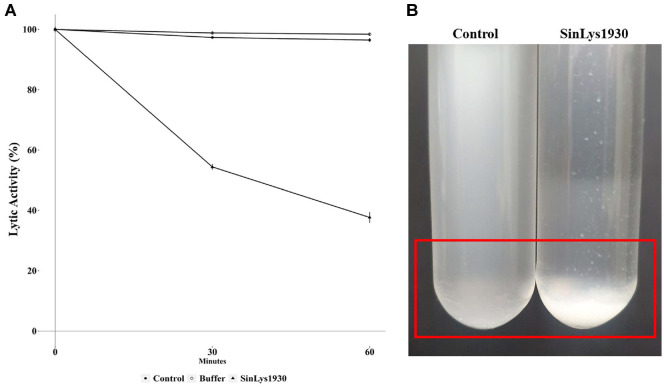
Lytic activity of SinLys1930 against *Streptococcus iniae* KCTC 3657 in sterilized seawater. (**A**) The reduction rate of optical density (%) is defined as the difference in OD_600nm_ from 0 to 60 min. Control, *S. iniae* resuspended in sterilized seawater (10 ml) without any addition; Buffer, *S. iniae* resuspended in sterilized seawater (9 ml) incubated with elution buffer without endolysin (1 ml); SinLys1930, *S. iniae* resuspended in sterilized seawater (9 ml) incubated with SinLys1930 (1 ml) under optimal conditions (final concentration, 1.96 μM; pH 6.0; 25°C; with Mg^2+^ treatment). (**B**) Lysis of *S. iniae* by SinLys1930 in sterilized seawater. Data are presented as means ± standard deviation of independent experiments conducted in triplicate. OD_600nm_, optical density at 600 nm.

The lytic activity of SinLys1930 was assessed against various bacterial species, including *Streptococcus* spp.(*S. parauberis* KCCM 43262, *S. sanguinis* KCTC 3284, *S. mutans* KCTC 3065, *S. alactolyticus* KCTC 3644, and *S. gallolyticus* subsp. *pasteurianus* KCTC 3878), *B. subtilis* KCTC 3014, *E. faecalis* KCTC 5191, and *E. coli* BL21 and DH5α. SinLys1930 demonstrated limited lytic activity against the tested streptococcal species, except for *S. parauberis*; SinLys1930 showed approximately 50% reduced lytic activity against *S. parauberis* KCCM 43262 relative to *S. iniae* KCTC 3657, while the lytic activity against other tested bacterial species was low, with relative activities below 20% (Fig. 4). Considering that *S. iniae* is a major causative agent of streptococcosis causing economic losses in aquaculture, we also assessed the lytic activity of SinLys1930 against the standard target strain *S. iniae* KCTC 3657 in seawater. SinLys1930 successfully decreased the optical density of *S. iniae* in sterilized seawater by ~60% after 1 h of incubation (Fig. 5A and 5B), demonstrating its potential as a non-antibiotic avenue controlling streptococcosis in aquaculture.

## Discussion

*S. iniae* is an increasingly significant pathogen that causes substantial economic losses in the aquaculture industry. Endolysins have been studied as promising antibacterial treatments, and recently, bioinformatics tools have enabled the identification of putative endolysin genes without the necessity of bacteriophage screening. Therefore, this study investigates a novel endolysin, SinLys1930, with potent lytic activity against *S. iniae* using bioinformatics tools.

Endolysins targeting gram-positive bacteria exhibit a modular structure comprising two active domains, namely an EAD at the N-terminus and a CBD at the C-terminus [9]. In the present study, amino acid sequence analysis using the NCBI CDD indicated that SinLys1930 has a modular structure comprising multiple domains with separate activities. The NlpC/p60 superfamily comprises endopeptidases that hydrolyze either the D-γ-glutamyl-meso-diaminopimelate or N-acetylmuramyl-L-alanine linkages present in the cell wall peptides. The NlpC/p60 superfamily generally consists of three α-helices and five β-sheet strands with three conserved regions, namely a cysteine residue at the N-terminus, a highly conserved glycine residue, and a histidine residue at the C-terminus [22]. Nucleophilic peptide bond attack is orchestrated by cysteine, while histidine plays a dual role in the catalytic process, acting first as a base and then as an acid catalyst to facilitate proton transfer. The NlpC/p60 domain of SinLys1930 also contain the three conserved motifs in the catalytic domain within an α+β fold architecture. The glucosaminidase superfamily, known as N-acetylglucosaminidases, cleaves the glycan component of PG on the reducing side of N-acetylglucosamine and the adjacent N-acetylmuramic acid. The CW-7 superfamily, encoded by the pneumococcal bacteriophage Cp-7, is located in the C-terminal region of the Cpl-7 lysozyme and contains a variable number (1 to 3) of repeated motifs (segments 39 to 42 amino acid-long) [23]. These CW-7 repeats consist of three-helix bundle folds that recognize and bind to N-acetyl-D-glucosaminyl-N-acetylmuramyl-L-alanyl-D-isoglutamine within the PG layer of the target bacteria. The conserved residues of the CW-7 repeats, including glycine, arginine, and various hydrophobic amino acids, facilitate their binding. These residues stabilize the enzyme through a combination of hydrophobic and polar interactions, forming shallow grooves that contain a binding site for muropeptides [24]. The CW-7 domain of SinLys1930 exhibits conserved amino acid regions and is predicted to adopt a three-helix bundle fold structure.

In the present study, recombinant SinLys1930 endolysin was successfully expressed and purified from the soluble fraction. We confirmed that the external application of the purified endolysin efficiently lysed the target bacteria. Therefore, turbidity reduction assays were performed to assess the optimal bactericidal activity of SinLys1930 against *S. iniae* and several other streptococcal species. SinLys1930 showed reduced activity around pH 8.0, which represents a limitation for direct application in natural seawater, where the pH typically remains relatively high [25]. In contrast, recirculating aquaculture systems often exhibit lower pH values due to carbon dioxide (CO_2_) accumulation from fish respiration, as dissolved CO_2_ forms carbonic acid and releases hydrogen ions [26, 27]. This pH-lowering effect is particularly prominent in high-density systems. Therefore, despite its limited activity at pH 8.0, SinLys1930 may still be suitable for use in recirculating aquaculture systems, where the environmental pH can fall within the enzyme’s optimal activity range. Fish are commonly categorized into three groups based on their preferred water temperature range; warm water species thrive in water temperatures ranging from 24 to 32°C, cool water species prefer temperatures between 18 and 24°C, and cold water species thrive in temperatures ranging from 13 to 18°C [28]. The concentration of NaCl in seawater is approximately 35 g per liter (600 mM) and can vary slightly depending on factors such as location, temperature, and climate. Taken together, our findings suggest that SinLys1930 could be used to target both cool and warm water species in seawater environments with slightly acidic conditions. Additionally, SinLys1930 exhibited a requirement for divalent metal ions, such as Ca^2+^ or Mg^2+^, to achieve optimal enzymatic activity. This metal ion dependence is a well-characterized feature of endolysins containing NlpC/p60 endopeptidases. A similar requirement for divalent cations was observed for Ecd09610CD1 against *C. difficile*, which contains the NlpC/p60 family of endopeptidases and glucosaminidases [29]. LyJH307, another example endolysin targeting *S. bovis*, also contains the NlpC/p60 family of endopeptidase domain, required Ca^2+^ for optimal activity, while Mg^2+^ treatment did not support this activity [30].

SinLys1930 exhibited a modular structure that incorporates two CBDs between the outer EADs, and this configuration is uniquely found in endolysins targeting streptococci [24]. Donovan and Frey (2008) developed the λSa2 endolysin, containing an N-terminal amidase-5 domain, C-terminal glucosaminidase, and two CW-7 domains between EADs, from the LambdaSa2 prophage [31]. The lytic activity of λSa2 endolysin was evaluated against *S. agalactiae* and other streptococcal species, and it exhibited higher lytic activity against *S. dysgalactiae* and S. pyogenes but lower lytic activity against *S. agalactiae* compared with other streptococcal species, despite being isolated from the *S. agalactiae* genome. In another study, an endolysin termed LySMP, whose structure is similar to that of SinLys1930, originating from a lytic bacteriophage of *S. suis* serotype 2, was developed and evaluated for its activity against various *S. suis* strains [32]. The LySMP endolysin exhibited higher lytic activity (30–60% decrease) against seven *S. suis* strains, but its activity was lower against the other strains, even though they were of the same species. Targeting the PG using the CW-7 domains reportedly accounts for Cpl-7 lytic activity against both gram-positive and -negative bacteria, however, Cpl-7 does not exhibit a broad-range lytic activity. The specificity of Cpl-7 is influenced by other factors that contribute to the definition of charge distribution and accessibility to PG, including capsular components, local PG composition, and surface-attached molecules [23]. Therefore, the differential lytic activity of SinLys1930 across streptococcal species might be attributed to other underlying factors that require further investigation.

Streptococcal infection in fish typically begins by affecting the skin, fins, gills, and other external organs, subsequently progressing to internal tissues and the blood [33]. The lytic activity of SinLys1930 against *S. iniae* in sterilized seawater demonstrated its potential application as an anti-pathogenic agent for aquaculture applications. Still, our *in vitro* turbidity might underestimate its therapeutic efficacy in actual aquaculture setting. This discrepancy between *in vitro* and *in vivo* efficacy has been observed with other endolysins, such as PlyD4, which demonstrated therapeutic efficacy against *A. hydrophila* infection in a zebrafish (*Danio rerio*) model. While *in vitro* turbidity assay showed only a 40% reduction in *A. hydrophila* turbidity, *in vivo* treatment with PlyD4 resulted in an 80% survival rate of the *A. hydrophila*-infected zebrafish [12]. There are multiple examples of endolysin applications for aquaculture as an anti-pathogenic agent. For instance, Gp110 from a *Salmonella* bacteriophage demonstrated a therapeutic activity against *A. hydrophila*-infected Nile tilapia (*Oreochromis niloticus*) [34], and Vplys60, produced by the *V. parahaemolyticus* bacteriophage qdvp001, also showed its considerable therapeutic efficacy for *V. parahaemolyticus*-infected *Artemia nauplii* [35]. Given the growing body of evidence supporting endolysin efficacy in aquaculture infection management, SinLys1930 represents a promising candidate for controlling streptococcal infections in fish. Further *in vivo* studies are warranted to fully evaluate its therapeutic potential and establish optimal treatment protocols for aquaculture applications.

## Supplemental Materials

Supplementary data for this paper are available on-line only at http://jmb.or.kr.



## References

[1] Vollset SE, Goren E, Yuan C-W, Cao J, Smith AE, Hsiao T (2020). Fertility, mortality, migration, and population scenarios for 195 countries and territories from 2017 to 2100: a forecasting analysis for the global burden of disease study. Lancet.

[2] Ahmad A, Abdullah SRS, Hasan HA, Othman AR, Ismail NI (2021). Aquaculture industry: supply and demand, best practices, effluent and its current issues and treatment technology. J. Environ. Manag..

[3] Sundberg L-R, Ketola T, Laanto E, Kinnula H, Bamford JK, Penttinen R (2016). Intensive aquaculture selects for increased virulence and interference competition in bacteria. Proc. Biol. Sci..

[4] Nachimuthu R, Royam MM, Manohar P, Leptihn S (2021). Application of bacteriophages and endolysins in aquaculture as a biocontrol measure. Biol. Control.

[5] Van Doan H, Soltani M, Leitão A, Shafiei S, Asadi S, Lymbery AJ (2022). Streptococcosis a re-emerging disease in aquaculture: significance and phytotherapy. Animals.

[6] Agnew W, Barnes AC (2007). *Streptococcus iniae*: an aquatic pathogen of global veterinary significance and a challenging candidate for reliable vaccination. Vet. Microbiol..

[7] Hegde A, Kabra S, Basawa RM, Khile DA, Abbu RUF, Thomas NA (2023). Bacterial diseases in marine fish species: current trends and future prospects in disease management. World J. Microbiol. Biotechnol..

[8] Gerstmans H, Criel B, Briers Y (2018). Synthetic biology of modular endolysins. Biotechnol. Adv..

[9] Wong KY, Megat Mazhar Khair MH, Song AA-L, Masarudin MJ, Chong CM, In LLA (2022). Endolysins against Streptococci as an antibiotic alternative. Front. Microbiol..

[10] Lai M-J, Lin N-T, Hu A, Soo P-C, Chen L-K, Chen L-H (2011). Antibacterial activity of *Acinetobacter baumannii* phage ϕAB2 endolysin (LysAB2) against both gram-positive and gram-negative bacteria. Appl. Microbiol. Biotechnol..

[11] Matamp N, Bhat SG (2019). Phage endolysins as potential antimicrobials against multidrug resistant *Vibrio alginolyticus* and *Vibrio parahaemolyticus*: current status of research and challenges ahead. Microorganisms.

[12] Wang C, Shi S, Wei M, Luo Y (2022). Characterization of a novel broad-spectrum endolysin PlyD4 encoded by a highly conserved prophage found in *Aeromonas hydrophila* ST251 strains. Appl. Microbiol. Biotechnol..

[13] Jiang Y, Xu D, Wang L, Qu M, Li F, Tan Z (2021). Characterization of a broad-spectrum endolysin LysSP1 encoded by a *Salmonella* bacteriophage. Appl. Microbiol. Biotechnol..

[14] Etobayeva I, Linden SB, Alem F, Harb L, Rizkalla L, Mosier PD (2018). Discovery and biochemical characterization of PlyP56, PlyN74, and PlyTB40-*Bacillus* specific endolysins. Viruses.

[15] Kim H, Seo J (2023). A novel strategy to identify Endolysins with lytic activity against methicillin-resistant *Staphylococcus aureus*. Int. J. Mol. Sci..

[16] Aziz RK, Bartels D, Best AA, DeJongh M, Disz T, Edwards RA (2008). The RAST server: rapid annotations using subsystems technology. BMC Genomics.

[17] Marchler-Bauer A, Derbyshire MK, Gonzales NR, Lu S, Chitsaz F, Geer LY (2015). CDD: NCBI's conserved domain database. Nucleic Acids Res..

[18] Drozdetskiy A, Cole C, Procter J, Barton GJ (2015). JPred4: a protein secondary structure prediction server. Nucleic Acids Res..

[19] Mirdita M, Schütze K, Moriwaki Y, Heo L, Ovchinnikov S, Steinegger M (2022). ColabFold: making protein folding accessible to all. Nat. Methods.

[20] Pettersen EF, Goddard TD, Huang CC, Meng EC, Couch GS, Croll TI (2021). UCSF ChimeraX: structure visualization for researchers, educators, and developers. Protein Sci..

[21] Lu Y, Wang Y, Wang J, Zhao Y, Zhong Q, Li G (2021). Phage endolysin LysP108 showed promising antibacterial potential against methicillin-resistant *Staphylococcus aureus*. Front. Cell. Infect. Microbiol..

[22] Anantharaman V, Aravind L (2003). Evolutionary history, structural features and biochemical diversity of the NlpC/P60 superfamily of enzymes. Genome Biol..

[23] Bustamante N, Campillo NE, García E, Gallego C, Pera B, Diakun GP (2010). Cpl-7, a lysozyme encoded by a pneumococcal bacteriophage with a novel cell wall-binding motif. J. Biol. Chem..

[24] Bustamante N, Iglesias-Bexiga M, Bernardo-García N, Silva-Martín N, García G, Campanero-Rhodes MA (2017). Deciphering how Cpl-7 cell wall-binding repeats recognize the bacterial peptidoglycan. Sci. Rep..

[25] Ezeanya N, Chukwuma G, Nwaigwe K, Egwuonwu C (2015). Standard water quality requirements and management strategies for fish Farming (A case study of Otamiri River). Int. J. Res. Eng. Technol..

[26] Datta S (2012). Management of water quality in intensive aquaculture. Respiration.

[27] Aslam SN, Navada S, Bye GR, Mota VC, Terjesen BF, Mikkelsen Ø (2019). Effect of CO_2_ on elemental concentrations in recirculating aquaculture system tanks. Aquaculture.

[28] Swann L. 1997. *A fish farmer's guide to understanding water quality*, Citeseer.

[29] Sekiya H, Yamaji H, Yoshida A, Matsunami R, Kamitori S, Tamai E (2022). Biochemical characterizations of the putative endolysin Ecd09610 catalytic domain from *Clostridioides difficile*. Antibiotics.

[30] Kim H, Lee HG, Kwon I, Seo J (2020). Characterization of endolysin LyJH307 with antimicrobial activity against *Streptococcus bovis*. Animals.

[31] Donovan DM, Foster-Frey J (2008). LambdaSa2 prophage endolysin requires Cpl-7-binding domains and amidase-5 domain for antimicrobial lysis of streptococci. FEMS Microbiol. Lett..

[32] Wang Y, Sun J, Lu C (2009). Purified recombinant phage lysin LySMP: an extensive spectrum of lytic activity for swine streptococci. Curr. Microbiol..

[33] Mishra A, Nam GH, Gim JA, Lee HE, Jo A, Kim HS (2018). Current challenges of *Streptococcus* infection and effective molecular, cellular, and environmental control methods in aquaculture. Mol. Cells.

[34] Bakiyev S, Smekenov I, Bissenbaev AK (2023). Comparative analysis of potential effects of three phage endolysins against antibioticresistant bacteria from the genus Aeromonas. Int. Aquatic Res..

[35] Srinivasan R, Chaitanyakumar A, Subramanian P, Mageswari A, Gomathi A, Aswini V (2020). Recombinant engineered phagederived enzybiotic in *Pichia pastoris* X-33 as whole cell biocatalyst for effective biocontrol of *Vibrio parahaemolyticus* in aquaculture. Int. J. Biol. Macromol..

