# Supplementary material for: Novel endolysin from Streptococcus iniae-Specific Prophage Selectively Inhibits Target Bacteria
Source: J Microbiol Biotechnol. 2025 Dec 29;35:e2508038. doi: 10.4014/jmb.2508.08038 (PMC12790987; doi:10.4014/jmb.2508.08038)
Supplement: Supplementary file 1 [file jmb-35-e2508038-supple.pdf]

J. Microbiol. Biotechnol. 2025. 35: e2508038

https://doi.org/10.4014/jmb.2508.08038

Supplementary Table

Novel endolysin from *Streptococcus iniae*-specific prophage selectively inhibits target bacteria

JoonBeom Moon[1†], Hanbeen Kim[1,2†], Suryang Kwak[3*], and Jakyeom Seo[1*]

[1]Department of Animal Science, Life and Industry Convergence Research Institute, Pusan National University, Miryang 50463 Republic of Korea

[2]Faculty of Land and Food Systems, The University of British Columbia, Vancouver, BC Canada V6T 1Z4

[3]Department of Bio and Fermentation Convergence Technology, Kookmin University, Seoul, 02707, Republic of Korea

[†]Both authors contributed equally to this work.

[*]Corresponding Authors:

Suryang Kwak, E-mail: skwak@kookmin.ac.kr

Jakyeom Seo, E-mail: jseo81@pusan.ac.kr

Table S1. Bacterial strains and growth conditions.

**Bacterial strain**	**Purpose**	**Growth conditions**[1]
Escherichia coli DH5α	Cloning host<br>Lytic spectrum	LB broth
Escherichia coli BL21 (DE3)	Expression host<br>Lytic spectrum	LB broth
Streptococcus iniae KCTC 3657	Indicator strain	BHI broth
Streptococcus parauberis KCCM 43262	Lytic spectrum	BHI broth
Streptococcus sanguinis KCTC 3284	Lytic spectrum	BHI broth
Streptococcus mutans KCTC 3065	Lytic spectrum	BHI broth
Streptococcus alactolyticus KCTC 3644	Lytic spectrum	BHI broth
Streptococcus gallolyticus subsp. pasteurianus KCTC 3878	Lytic spectrum	BHI broth
Bacillus subtilis KCTC 3014	Lytic spectrum	BHI broth
Enterococcus faecalis KCTC 5191	Lytic spectrum	BHI broth

LB, Luria–Bertani; BHI, brain heart infusion.

[1]All bacterial strains used in this study were grown at 37°C.
